# A feasibility study of an intervention for structured preparation before detoxification in alcohol dependence: the SPADe trial protocol

**DOI:** 10.1186/s40814-019-0446-1

**Published:** 2019-04-27

**Authors:** Christos Kouimtsidis, Ben Houghton, Heather Gage, Caitlin Notley, Vivienne Maskrey, Allan Clark, Richard Holland, Anne Lingford-Hughes, Bhaskar Punukollu, Theodora Duka

**Affiliations:** 1 Research and Development, Abraham Cowley Unit, Surrey and Borders NHS Foundation Trust, Surrey, Chertsey, KT16 0AE UK; 2 University of Surrey, 388 Stag Hill, Guildford, GU2 7XH UK; 3 Norwich Medical School, University of East Anglia, Norwich Research Park, Norwich, NR4 7TJ UK; 4 University of East Anglia, Norwich Research Park, Norwich, NR4 7TJ UK; 5 University of Leicester, University Road, Leicester, LE1 7RH UK; 6 Imperial College London, Burlington Danes Building, Hammersmith Campus, 160, Du Cane Road, London, W12 0NN UK; 7 Camden and Islington NHS Foundation Trust, St Pancras Hospital, 4 St Pancras Way, Kings Cross, London, NW1 0PE UK; 8 University of Sussex, Falmer, Brighton, BN1 9RH UK

**Keywords:** Alcohol dependence, Detoxification, Structured preparation, SPADe

## Abstract

**Background:**

Alcohol-related harm is currently estimated to cost the National Health Service (NHS) in England £3.5 bn a year. Of the estimated 1.6 million people with some degree of alcohol dependence, some 600,000 are believed to be moderately or severely dependent and may benefit from intensive treatment. Outcomes from medically assisted withdrawal, also referred to as detoxification, are often poor, with poor engagement in relapse prevention interventions and subsequent high relapse rates. Detoxification is costly both financially and to the individual. It has been found that people who experience multiple detoxifications show more emotional and cognitive impairments. These changes may confer upon them the inability to resolve conflict and increased sensitivity to stress thus contributing to increased vulnerability risk of relapse. The study aims to test the feasibility of using a group intervention aiming to prepare participants for long-term abstinence before, rather than after, they have medically assisted detoxification. The current study will establish key parameters that influence trial design such as recruitment, compliance with the intervention, retention, and sensitivity of alternative outcome measures, in preparation for a future randomised controlled trial (RCT). This paper presents the protocol of the feasibility study.

**Methods:**

The study corresponds to phase 2 of the Medical Research Council (MRC) complex interventions guidelines which cover the development and feasibility testing of an intervention. The work is in three stages. The development, adaptation and implementation of the Structured Preparation before Alcohol Detoxification (SPADe) intervention (stage 1), a randomised feasibility study with economic evaluation (stage 2) and a qualitative study (stage 3). Fifty participants will be recruited from two community alcohol treatment services in England. Participants will be randomised in two arms: the treatment as usual arm (TAU), which includes planned medically assisted detoxification and aftercare and the intervention arm in which participants will receive structured group preparation before detoxification in addition to TAU. The main outcomes are duration of continuous abstinence with no incidents of lapse or relapse, percentage of days abstinent and time to relapse.

**Discussion:**

The socioeconomic harms associated with alcohol have been well-documented, yet existing treatment options have not been able to reduce high relapse rates. This study will build on existing naturalistic studies underpinned by psychological interventions offered early and before detoxification from alcohol, which aim to reverse automatised habitual behaviours and thus may help us to understand how better to support people to remain abstinent and improve post detoxification outcomes.

**Trial registration:**

ISRCTN, 14621127; Registered on 22 Feb 2017

## Background

Tackling the impact of harmful and dependent drinking is a key global public health priority. Harmful drinking refers to consuming alcohol at amount and patterns that would affect the person’s physical and mental health and lead to personal and social harm. The harm is linked to both chronic as well as episodic heavy use (intoxication). For example, global estimates suggest that one in five adults report at least one occasion of heavy episodic drinking in the past month [1]. Alcohol harmful use is linked directly to a range of health disorders, including high blood pressure, heart disease, stroke, liver disease, some cancers and depression [2]. Furthermore, more than 21,000 people died from alcohol-related causes in England in 2012 [2]. As far as financial cost is concerned, estimates suggest that alcohol-related harm (from harmful and dependent use) costs the National Health Service (NHS) in England £3.5 bn a year [2].

Despite the fact that the prevalence of dependent drinking is far less than that of harmful drinking, of an estimated 1.6 million people with some degree of alcohol dependence, some 600,000 are believed to be moderately or severely dependent and may benefit from intensive treatment [3]. Individuals who are ‘moderately’ or ‘severely’ dependent represent the severe end of the spectrum of alcohol use disorders and consume alcohol at levels that are likely to have a severe impact on their own health and mortality, the health and behaviours of others (family members) and to have economic and social implications [4]. Alcohol dependence impacts significantly on the NHS, as individuals affected are at increased risk of accidents, and more likely to attend an accident and emergency department (A&E) and have emergency admissions [2, 5]. In the longer term, alcohol-dependent individuals are more likely to suffer from chronic conditions requiring expensive treatment, such as liver and heart disease [2].

Current treatment guidelines suggest that treatment for moderate to severe alcohol dependence needs to be planned, with up to four motivational sessions focusing on treatment engagement and development of aftercare support, followed by medically assisted withdrawal (also referred to as detoxification) and aftercare support [4]. Benzodiazepines are normally prescribed during detoxification to reduce the overt symptoms of alcohol withdrawal (sweats, tremor) as well as potentially life-threatening complications (e.g. convulsions, delirium tremens) [4]. However, these drugs do not prevent alcohol craving, relapse back into alcohol drinking and other long-term effects on mental functioning [4, 6]. Outcomes from detoxification are often poor with low proportions engaging in aftercare [7] and high relapse rates [4]. Repeat detoxification is costly. Additionally, there is some evidence suggesting that people with alcohol dependence who experience multiple detoxifications show increased impairment in mental functioning [8, 9]. It is important to clarify that these changes are shown to be associated, not caused by the detoxification process. Nevertheless, they may confer inability in conflict resolution and increased sensitivity to stress, both of which may contribute to relapse and might compromise the effectiveness of aftercare support [8–11]. There is also evidence to suggest that multiple detoxifications can exacerbate craving, adversely impacting on subsequent attempts at achieving abstinence [12].

In the light of the abovementioned evidence indicating the potential risk of an accumulation of adverse effects following repeated detoxifications, and until definitive evidence is generated to confirm or refute a causal relationship, it is prudent to maximise treatment effectiveness and to reduce the risk of exposing people to repeated detoxifications and the potential adverse effects associated with the detoxification process itself. Although the existing evidence is only indicative, it is possible that the long-term course, and economic and social impact of alcohol dependence, is not only due to the natural process of the phenomenon, but is also associated with the existing treatment approach that emphasises the importance of the provision of detoxification per se, without any emphasis on long-term sustainable outcomes. There is currently no guidance specifically on preparation for the detoxification process, apart from general guidance on care coordination and case management [4]. Absence of specific guidance reflects the lack of developed interventions in this area. Evidence is required on whether structured preparation before detoxification rather than detoxification alone improves treatment outcomes and therefore whether the whole treatment paradigm should shift. Before embarking on a full trial of the effectiveness of such structured preparation, there is a need to undertake a feasibility study to establish key parameters that influence trial design such as recruitment, adherence to the intervention, retention and sensitivity of alternative outcome measures.

A literature search of PubMed Central using alcohol relapse prevention, treatment-related MeSH terms undertaken in June 2014 found (1) that group interventions with diverse theoretical bases are considered to be more cost-effective than one-to-one interventions [13] and (2) cognitive behaviour therapy (CBT) relapse prevention interventions are well-supported by evidence [14].

In the UK, an innovative group intervention for preparation before alcohol detoxification, based on CBT relapse prevention interventions, developed by members of the research team, reduced detox dropouts [15] and improved outcomes at 1, 3 [5] and 6 months [16]. However, these findings were from small naturalistic studies. Qualitative evidence found that ‘regaining control’ was the main learning point across all group sessions of the programme [17].

This proposed feasibility study builds on the above preliminary evidence and aims to refine the preparatory intervention and assess the feasibility of conducting a large-scale evaluation (Structured Preparation before Alcohol Detoxification (SPADe)) for people with moderate to severe alcohol dependence, as an adjunct to usual care, consisting of planned detoxification and aftercare.

The intervention under investigation in this study is based on Plans, Responses, Impulses, Motives, Evaluations (PRIME) theory of motivation [18] and learning theories [19] that underlie the use of psychological interventions aiming to reverse the development of automatised behaviour and associated loss of control, such as CBT relapse prevention interventions. It combines the long-established (in alcohol treatment) ethos of group intervention and follows the biological principle of homeostasis, which is disturbed with prolonged alcohol use [20], in order to help individuals to regain control over drinking as the first step towards lifelong sustainable abstinence.

## Aims and objectives

The key research question is can we design a large-scale, randomised controlled trial (RCT) that will answer whether SPADe as an adjunct to usual care is more effective than usual care alone in helping adults to maintain longer periods of alcohol abstinence? The feasibility trial will compare the use of SPADe as an adjunct to treatment as usual with treatment as usual in the participating sites.

Furthermore, reduction in subjective measures of alcohol dependence and craving as well as improvement in objective measures of mental functioning will be explored. Finally, we will conduct qualitative interviews with service users, their carers and service providers, to assess the acceptability of the treatment and to explore their experience of the treatment including any barriers and/or facilitators to taking part in the study. Findings from these interviews will enable us to refine the SPADe intervention and the design of the future definitive RCT.

## Methods/design

The study corresponds to Phase 2 of the Medical Research Council (MRC) complex interventions guidelines, which guides the development of an intervention [21]. Therefore, the study has three stages:

### Stage 1—adaptation and implementation of the intervention

We will adapt the existing pre-alcohol detox group protocol [6, 16, 17] by incorporating feedback from service users and group facilitators gathered at two patient and public involvement (PPI) meetings to produce a manual for the intervention. Group facilitators will be recruited from existing staff and trained in the intervention via the use of the manual. When training is complete, we will implement the SPADe groups across our two study sites.

A 1-day training event will take place in each participating site and one central event for those not able to attend locally. At least six members of staff will be trained (to allow cross-cover for sickness and leave). Facilitators will have face-to-face or Skype supervision at least monthly or more frequently if required weekly by the chief investigator.

Importantly during this phase, treatment as usual procedures will also be standardised across the sites. To reduce the risk of contamination between the intervention and the control arms of the study, the trained intervention group facilitators will offer only the intervention and they will not undertake individual work with any service user for the period of the study. They will also provide the one-to-one version of the intervention to non-English speaking service users (via an interpreter if required). The rest of the staff will provide the treatment as usual.

### Stage 2—feasibility study

#### Design

This is a single-blind (outcome assessor blind to allocation), parallel, two-arm, feasibility RCT comparing the clinical effectiveness of SPADe as an adjunct to treatment as usual (TAU), against TAU. Participants will be assessed at baseline, 3, 6 and 12 months following randomisation. Additionally, qualitative interviews will be carried out at 3 and 9 months (see stage 3 below).

#### Setting

Participants will be recruited from specialist alcohol community services offering recovery-orientated treatment for people with alcohol use disorders (AUD). Recruitment will take place in two sites, both offering the intervention and the treatment as usual, in order to explore challenges associated with implementation of the intervention across a range of services.

#### Participants

**Fig. 1:**
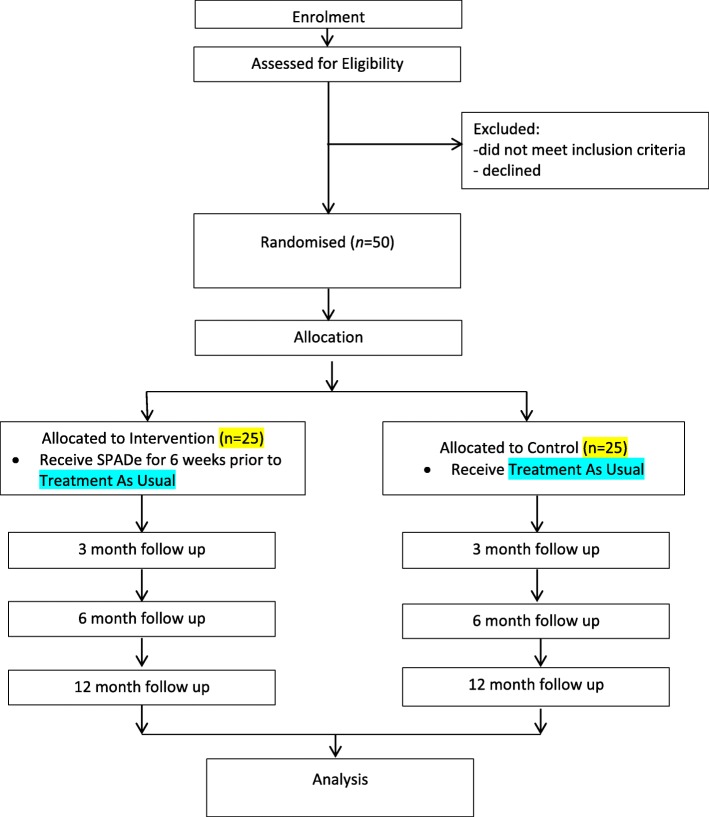
Recruitment and follow-up flow diagram

We will recruit 50 alcohol-dependent participants, aged 18 or over who have a desire to stop drinking. As both recruiting services operate within a multi-lingual, multi-faith and multi-cultural environment, we will provide study information in the two most prevalent languages spoken by non-English speakers in our locations, in addition to the material in English. Should we recruit any non-English speaking participants to the intervention arm, the sessions will be provided in one-to one format by the intervention group facilitator, with the support of an interpreter if required. Participant flow to the study is shown in Fig. 1. We will aim to recruit at least one of the above participants in the qualitative study to explore their experience.

#### Recruitment

When entering the service, eligible service users will be advised about the study by the clinician undertaking the initial assessment, following the completion of the assessment. If interested, the service user will be given the participant information sheet and a member of the research team will contact them a few days later to ascertain if they would like to be recruited into the study. An arrangement will be made to meet in the clinic to take informed consent and collect baseline information. Participants will receive a £10 shopping voucher (not valid for alcohol purchase) per completion of each research assessment throughout the trial (four in total, with additional two vouchers if they take part in the qualitative study, see below) (see Fig. 1).

#### Inclusion criteria

Inclusion criteria are as follows:

1. Presentation to either of the two alcohol services seeking abstinence from alcohol.
2. Alcohol dependence (moderate to severe), scoring 16 and above on Severity of Alcohol Dependence Questionnaire (SADQ) (see the ‘Outcome measures’ section below). This level of dependence indicates that it would be clinically appropriate to receive a medically assisted detoxification [4].
3. Stated intention to stay in the area within the time period of the intervention.
4. Willingness to be part of a group intervention if randomised to receive it.

#### Exclusion criteria

Exclusion criteria are as follows:

1. Age less than 18 (as not usually treated by specialist alcohol services).
2. Pregnancy: pregnant women need urgent intervention to withdraw from alcohol, due to the effect of alcohol on the foetus.
3. Known terminal illness with life expectancy of less than 6 months.
4. Severe medical condition that requires urgent medical admission, which would lead to an unplanned medically assisted withdrawal.
5. Severe cognitive impairment that compromises capacity and/or ability to participate in a group intervention.
6. Acute stage of severe and enduring mental illness (schizophrenia, bipolar affective disorder, recurrent depressive disorder—current episode severe), when acute symptomatology compromises service user’s ability to participate in a group intervention.

#### Trial arms

##### Treatment as usual

Usual detoxification/control comprises planning for detoxification, detoxification delivery and aftercare. Standardised procedures across sites will be confirmed in phase 1. Service users enter detoxification at the first available opportunity (likely to be within 4 weeks from presentation). Whilst waiting for detoxification, they will meet their key worker (one-to-one) on 3–4 occasions to maintain motivation and plan aftercare.

Detoxification is medically assisted in the community as an outpatient, or inpatient, as clinically indicated. The choice depends on health risk factors and availability of social support during detoxification. The type of detoxification has been shown by NICE not to affect treatment outcomes [4]. Aftercare (following detoxification) includes peer support groups such as Self-Management and Recovery Training (SMART) Recovery or Alcoholics Anonymous (AA), a small number of individual key worker sessions, pharmacological interventions as appropriate or more comprehensive aftercare group programme. Participants in both intervention and control groups will receive all the above elements of usual care available in the recruiting service. Each participant’s care pathway will be recorded in detail and analysed for variability within and between sites as part of the economic evaluation (see below), to ensure these are equivalent across trial arms.

##### Intervention

The intervention provides structured group preparation (additional to usual care) with the aim of helping participants regain control over their drinking, prior to detoxification. Part of this control is lifestyle modification, which is necessary for maintaining abstinence post detoxification. Regaining control is linked with developing new coping skills and enhanced self-efficacy.

The six sessions are numbered and offered weekly in a given order. Stabilisation of the amount and pattern of drinking is a common theme across all the sessions. To that effect, each session can act as an entry point (i.e. an open rolling programme group), despite the special theme covered in depth during the second part. Each session has two facilitators, lasts for 1 h and is divided into three parts:

- In the first part (15 min), group rules are established (as advised in PPI meetings), new members are introduced, aims of the intervention, in-between sessions practice allocated in previous session reviewed as are individual targets set for the previous week are reviewed.
- The second part (30 min, main part) explores the following themes depending on the session number: 1—understanding habit, addiction and alcohol dependence; 2—stabilise and control your drinking; 3—lifestyle changes for you and the people around you; 4—reduction of your drinking; 5—achieving abstinence; and 6—relapse prevention strategies.
- In the third part (15 min), the group summarises the main learning points and agrees in-between sessions practice and targets to be achieved before the next session. A group work folder will be provided enabling notes and worksheets to be kept together, as also suggested by PPI meetings.

The number of participants per group at any point is between two and eight, as this is considered appropriate for theory-based treatment groups, to reach a balance between education, treatment, group interactions and facilitator’s attention to each participant [13]. This maximum is unlikely to be reached in the feasibility trial. If only one participant is present, the session will be offered by one of the facilitators as an individual session. The duration of 1 h as suggested in PPI, will reduce risk of withdrawal symptoms and help participants to maintain concentration.

#### Outcome measures

Feasibility outcomes will be (1) Recruitment and retention rates: we will monitor (monthly) the number of alcohol-dependent clients accessing services during the recruitment period of the study, how many meet the eligibility criteria and how many were invited and accepted into the study and retained in each group for the full 12 months. (2) Compliance with treatment: number of SPADe sessions attended (for the intervention arm) using the facilitator’s record of attendance. (3) Data collection and completeness: attendance for assessments and completeness of instruments; loss to follow-up and missing data for all outcomes will be analysed.

A variety of possible outcomes will be used, assessed at different time points (3, 6 and 12 months following randomisation), so the primary outcome for the main trial can be identified and sample size calculations conducted, including duration of continuous abstinence with no incidents of lapse or relapse; percentage of days of abstinence (PDA) (both self-report using Timeline Followback method (TLFB)) [22]; and time to relapse (from stopping alcohol to first day of alcohol use, also as defined by self-report).

Secondary outcomes will be measured using validated instruments wherever possible at the same time points as above:

1. Severity of Alcohol Dependence Questionnaire (SADQ), a 20-item self-completion questionnaire, scores range 0 to 60 (16 to 29 indicates moderate severity, above 30 severe dependence) [23].
2. Alcohol Urge Questionnaire (AUQ), an eight-item self-completion questionnaire containing three domains of drinking urges: desire for a drink, expectation of positive effect from drinking and inability to avoid drinking [24].
3. Incentive conflict task (ICT). We have introduced the ICT as incentive conflict task (ICT), a newly developed task which models contributors to relapse (inherent conflict in abstaining drinkers between the intention to abstain from drinking, and the desire to drink) [9]. The ICT requires experimental subjects to abstain from responding during the presentation of a novel compound stimulus made up of two visual cues that she/he has previously learned to signal reward availability when presented separately. Thus, the task requires the subject to respond for the monetary reward, but to withhold responding under conditions in which an increased size of reward (both positive cues together) might be reasonably anticipated. In other words, ICT engages both bottom-up triggers of reward-seeking and the top-down processes that normally modulate and veto responses to such triggers. We have suggested that the task thus creates a conflict between abstaining and responding for reward similar to that experienced by the patients before relapse and that the impaired ability of multiple-detoxified patients to perform the task accurately reflects the consequences of detoxification on top-down control of their behaviour. Alcohol-dependent patients, as they experience successive detoxifications and their alcohol dependence increases, become increasingly impaired in performing the ICT [25].
4. Euro-QoL (EQ-5D-5 L); English version [26], which is a short questionnaire assessing general healthcare used in economic evaluations for the calculation of quality-adjusted life years.
5. Self-reported participation in aftercare activities, using a specifically developed log, measuring type and frequency of activity attended, during the period prior to the follow-up interview.

All instruments will be administered by research assistants in face-to-face assessments with participants. These assessments will take place at a convenient location for each participant, including their home. Research assistants (RA) will be fully trained to administer all outcome measures (see Table 1 below).

**Table 1:** The use of instruments at different time points

Instruments	Baseline	3 months	6 months	12 months
TLFB (previous 90 days)	v	v	v	v
SADQ	v	v	v	v
AUQ	v	v	v	v
ICT	v	v	v	v
EQ-5D-5 L	v	v	v	v
Self-reported participation in aftercare		v	v	v

#### Randomisation and masking

Once consented, participants will be randomised using a third-party web-based randomisation system which will ensure concealed allocation. Participants will be stratified according to the number of previous detoxifications (> 2 vs ≤ 2) and site. Randomisation will have a random block size [2–4]. Research assistants will be blind to the randomisation of the opposite study site and will conduct those follow-up interviews. Any breach in blinding/unmasking, a common problem with single-blind trials, will be monitored by asking the research assistants to report any such incident, to ensure that the research assistants are not aware, as far as possible, to which group the individual was allocated in the opposing study site.

#### Treatment fidelity

Twenty-five percent of the intervention sessions, offered across both recruitment sites, during the whole duration of the study recruitment and treatment stage, will be observed and rated using the Yale Adherence and Competence Scale (YACS II) (2005) [27]. This is a validated instrument measuring the use of CBT techniques. The tool can be adapted to the focus of the therapy under investigation. Furthermore, 10% of the sessions will be observed and rated by an additional independent rater using YACS II. Group facilitators will complete a self-assessment form following each session to reflect on their fidelity to the intervention manual.

Twenty-five percent of the key-working sessions offered to the control group will be audio recorded and be rated to assess possible contamination between the study arms using a specifically developed form based on YACS II items as well as the main objectives of the SPADe group intervention. Ten percent of those sessions will also be independently rated.

#### Statistical analysis

The main analysis will be based on the intention-to-treat principle considering all randomised clients according to the arm they were allocated. As the trial is a feasibility study, the main analysis is to be descriptive and estimate the potential effect sizes and parameters required for the sample size calculation, rather than formal hypothesis testing.

The feasibility outcomes will be summarised using descriptive statistics where appropriate. The potential primary and secondary outcomes will be summarised by arm, as well as completion rates estimated for each outcome measure. Duration of continuous abstinence, as measured from randomisation, and the time to relapse, as measured from the end of the intervention, will be analysed using a Kaplan-Meier curve and log-rank test. The percentage of days of abstinence (PDA) will be analysed using a regression model. Formal hypothesis tests and confidence intervals will be conducted although due to the nature of this trial, they will be treated cautiously and the main focus will be on the completion rate of outcomes and the estimation of parameters required for a sample size calculation for the main trial.

#### Economic evaluation and analysis

The economic evaluation that will run alongside the future trial will assess the cost-effectiveness of the SPADe intervention, compared to usual care, from the NHS and wider public service perspective. Economic evaluation is required in the feasibility study to inform decisions about how costs and outcomes would be measured in any future definitive trial.

The feasibility study will seek to gain an understanding of the main resource items for which comprehensive data collection would be required in the main trial. The resources involved in delivering the intervention will be collected from providers and will cover human resources, facilities, materials and overheads, so that full economic costs and average costs per participant can be calculated. Usual care (received by both arms) may vary. Hence, data on care provided will be collected on an individual client basis from clinic records so that variability can be explored both within and between groups.

Alcohol dependence can give rise to significant use of health, social and criminal justice services. The cost of implementing SPADe may be offset if it results in increased numbers of successful detoxifications and consequent reductions in alcohol-related utilisation of these services. To capture this possible effect, data on service utilisation will be gathered retrospectively from participants at 3-, 6- and 12-month assessments by self-report. The Client Service Receipt Inventory, customised in collaboration with service users, will be used for this purpose. Included items will cover GP, mental health services, specialist alcohol services, A&E attendance, inpatient stays and contact with social and criminal justice services. Items of service use will be converted to costs (British pounds, 2017) using nationally validated sources [28, 29] and national reference costs for secondary care (https://www.gov.uk/government/publications/nhs-reference-costs-2012-to-2013). Service use data will be analysed descriptively to assess validity.

The feasibility study will also explore the properties of the alternative primary outcomes to assess their value as measures of effectiveness for the full economic evaluation. The health-related quality of life outcome for the economic evaluation will be EQ-5D-5 L, measured at each assessment point, from which a standardised unit of measurement—the quality-adjusted life year (QALY)—can be obtained. A preliminary cost-effectiveness analysis will be undertaken to determine the likely advantage of conducting a full economic evaluation. Total and per participant costs will be compared between groups to assess whether overall there are cost savings associated with the SPADe intervention, compared to usual care, and how these compare with client outcomes.

### Stage 3—qualitative study

#### Aim

The qualitative study seeks to understand the experiences of the feasibility trial participants. Although we are interested in individual interpretations of experiences, recognising that there are many views on the world all with equal ‘validity’, a critical realist approach has been chosen as we also seek to uncover practicalities, understand key events and important aspects specifically of trial procedures (recruitment and randomisation, for example, and then specific aspects of either the intervention or of treatment as usual), from the perspectives of individuals involved in the study, that may inform our approach for a definitive trial.

#### Method

We will use individual interviews, as being the most appropriate means of assessing participant experience guided by specific topics and outcomes of interest. Thematic coding of all qualitative interview data will be undertaken, to draw out participant perspectives that illuminate trial procedures to inform our future approach to evaluation [30]. This is an approach that we have successfully employed in trial feasibility work in the past [31].

#### Sampling

A purposive sample of participants across both trial groups and areas (approximately *n* = 20) will be interviewed at 3 months to establish experiences of randomisation, recruitment and initial trial procedures. These early interviews (wave 1) will select participants by accessing demographic details and purposefully selecting individuals across a range of variables, including age, employment status, length of time in treatment, and severity of dependence; aiming for maximum variation in the sample. Further variables may be selected for depending on the specific features of individuals recruited to the study and in consultation with the project steering group (including PPI input). Follow-up participant interviews with the same interviewees will be undertaken at 9 months (completion of the study, wave 2) to give specific feedback on retention issues and treatment conditions (specifically in relation to the control condition or receiving the intervention).

#### Data collection

Interview guides will be developed in consultation with PPI representatives and following piloting, to capture the range of possible views, but will be flexibly employed to allow participants to discuss experiences and opinions in a way that is meaningful to them. Thus, although interviews are semi-structured and focused very much on trial procedures to inform a future definitive study, we will also consider the lens of individual experience and understanding in the interpretation of our findings. Qualitative interviews will be undertaken by the study RA, who will be trained in qualitative interviewing techniques. Qualitative interviews will be undertaken at treatment premises and timed to coincide with appointments, where possible, to minimise inconvenience to the participant. All interviews will be audio recorded following informed consent. A £10 shopping voucher will be offered to participants as compensation for their time commitment per interview they attend (total of two vouchers). All interviews will be transcribed verbatim, at which stage transcripts will be fully anonymised. Identifying participant details for follow-up (wave 2) interviews will be stored on password-protected encrypted computers, accessible only to members of the study team. Anonymised transcripts will be password-protected, stored separately, and will be archived for potential secondary analysis at later date. Archived transcripts will be kept in fully anonymised form, conforming to data archiving procedures such as those of the ESRC UK data archive.

#### Triangulation

A selected sample of carers and staff involved with the intervention will be interviewed on study completion. We anticipate that 5–6 carer interviews and 5–6 staff interviews will be sufficient to inform our aim of informing study procedures and will add to perspectives of participant experience. Carers and staff will not receive any monetary compensation for participating in the study. Thus, these other views will help us to triangulate the qualitative data and offer a range of different perspectives that may be useful for meeting our feasibility aims. For example, staff may usefully offer practical service-based recommendations for future trial procedures. Carers will offer a unique perspective on how participants experience the intervention which may give an alternative view for our consideration. The topic guides for these carer and staff interviews will be finalised following completion of the participant data collection, in order that emergent analysis can shape areas for further discussion with staff and carers. Contextual factors will be included such as staff descriptions and perceptions of service delivery (local level contextual factors). Relevant local and national policy will be monitored for the study duration (macro-level contextual factors).

#### Analysis

Qualitative analysis will thematically compare key experiences between trial groups (https://www.gov.uk/government/publications/nhs-reference-costs-2012-to-2013) and consider staff experiences across trial areas. Analysis will be discussed at regular meetings to develop the thematic coding scheme and will utilise service user perspectives in reaching consensus. Analysis will be led by one study RA and supervised by CN, who will undertake independent coding to triangulate perspectives and ensure that interpretations are reliable and valid. We consider that our proposed sample size of 20 participants will be adequate to meet project aims and to achieve ‘saturation’ of coding in terms of the range of likely experiences and views. However, we will continue to sample in an iterative manner should our emergent analysis suggest that saturation is not yet reached. Final analysis will offer in-depth qualitative explanation of the quantitative study findings. Qualitative analysis findings will therefore inform feasibility questions considered by the trial steering group and enable a thorough evaluation of the process of the feasibility study in order to proceed to a definitive study.

### Ethical and research and development reviews

The study was approved by the Health Research Authority (HRA) Research Ethics Committee (REC) (IRAS ID: 213086) for all participating centres. All study personnel will comply with the MCA 2005 [32] and published research governance guidelines. We anticipate that all participants will have the capacity to consent and sufficient verbal communication skills to take part in the treatment and in the qualitative interview. Informed written consent will be obtained from all participants. As this is a non-invasive intervention, we do not anticipate any adverse events, but we will follow safety reporting guidance issued by the National Research Ethics Service (UK) for studies except clinical trials of investigational medicinal products. The study has been approved by the research and development (R&D) departments of the relevant recruiting sites.

### Patients and public involvement

The first PPI event was organised in 2014. Advice had been sought from three service users who had completed the groups and another service user who did not. Suggestions included being able to ‘write things down’ and keep in a group work ‘folder’ as well as preference for face-to-face research assessments. Feeding back results to service users via service user reps, newsletters, and peer mentors and on the electronic screen display in service waiting rooms was also requested and implemented. The concept of randomisation was met with enthusiasm by service users and it was felt that people would be happy to participate in the study. All suggestions were incorporated in the study protocol and dissemination plans. There were two discussion groups with staff at two treatment centres where the need for research outcomes and the practicalities of running trials was met with eagerness by staff.

A second PPI event took place in 2015 at the service users’ forum where the feasibility study design was discussed. Suggestions were made about the provision of written information about the group, the importance of rules for acceptable behaviour during the group sessions, the maximum number of sessions and participants and the aftercare planning as part of preparation. One service user expressed interest in being a collaborator in the study, and he became part of our study steering committee. Following the PPI event, existing information about the group was reviewed, modified and approved by the service users’ forum.

## Discussion

The SPADe trial builds on previous work completed by the authors, which has shown that a structured preparation approach prior to alcohol detoxification can improve overall capacity of the treatment pathway by increasing patients’ engagement and by reducing dropout rates from detoxification [6, 15]. Two open studies indicated that the intervention improves outcomes (continuous abstinence) at 3 [15] and 6 months [16]. A qualitative study with service users who have completed the intervention [17] and a process study [33] have indicated that the theory-based intervention works as expected and predicted by the underpinning theory.

This feasibility trial will help us to answer questions of acceptability of the intervention by wider group of service users, carers and service providers. It will provide evidence on implementation issues such as the training, supervision and overall support required to implement the intervention. In more detail will enable us to do the following:

1. Measure the number of eligible participants and those not, willingness of clinicians to recruit participants, recruitment rate, loss to follow-up, adherence to the intervention and standard deviation of the primary outcome measures. This will ultimately inform the sample size calculation for a multi-centre clinical trial.
2. Determine the acceptability of randomisation to service users, through its effect on recruitment, dropout rates and via qualitative interviews.
3. Determine, through response rates to questionnaires, the appropriateness and the acceptability of the outcome measures to service users, in order to explore the suitability of our chosen secondary outcome measures, that is, percentage of days of abstinent, service use and health-related quality of life.
4. Estimate the time needed to collect and analyse baseline and outcome data
5. Explore the utility of the health-related quality of life instrument (EQ-5D-5 L) (see the ‘Outcome measures’ section) in allowing the estimation of quality-adjusted life years in the sample.

The development of the intervention manual, fidelity checks (standardised measures and self-reflection tools scored by two assessors, one an independent) and quality monitoring of the TAU key-working sessions are consistent with the recommendations of the Treatment Fidelity Workgroup of the NIH Behaviour Change Consortium [34]. This group recommended five broad areas in which fidelity could be enhanced during clinical trials: study design, training of treatment providers, delivery of treatment, receipt of treatment and enactment of treatment skills. Specific suggestions to avoid threats to fidelity included the following: development of a treatment manual that includes information about treatment dose (length and number of contacts) and specific content of each contact, standardisation of therapist training, monitoring the intervention with fidelity checklists and inclusion of strategies to measure the recipient’s comprehension and enactment of the intervention principles addressed.

The feasibility RCT will provide support for the design and execution of a future full RCT by testing different primary and secondary outcomes at different time points and should allow estimation of the sample size required. In addition, the use of ICT will explore further the potential harm minimisation role of structured preparation prior to detoxification by improving cognitive functioning. Finally, the collection of data regarding aftercare treatment engagement will provide further evidence on the potential positive role of our structured preparation in aftercare participation and the associated exposure to the psychosocial treatment ingredients recommended by current treatment guidelines.

### Trial status

At the time of the manuscript submission, the trial has started recruitment of participants. At the time of manuscript acceptance, recruitment was completed (June 2018).

## Abbreviations

A&E: Accident and emergency department
AA: Alcoholics Anonymous
AUD: Alcohol use disorders
AUQ: Alcohol Urge Questionnaire
CBT: Cognitive behaviour therapy
HRA: Health Research Authority
ICT: Incentive conflict task
MRC: Medical Research Council
NHS: National Health Service
PDA: Percentage of days abstinent
PPI: Patient and public involvement
PRIME: Plans, Responses, Impulses, Motives, Evaluations
RA: Research assistant
RCT: Randomised controlled trial
REC: Research Ethics Committee
SADQ: Severity of Alcohol Dependence Questionnaire
SMART: Self-Management and Recovery Training
SPADe: Structured Preparation for Alcohol Detoxification
TAU: Treatment as usual
TLFB: Timeline Followback method
YACS: Yale Adherence and Competence Scale
